# Effect of vital dyes on human corneal endothelium and elasticity of Descemet’s membrane

**DOI:** 10.1371/journal.pone.0184375

**Published:** 2017-09-13

**Authors:** Isabell P. Weber, Mrinal Rana, Peter B. M. Thomas, Ivan B. Dimov, Kristian Franze, Madhavan S. Rajan

**Affiliations:** 1 Department of Physiology, Development and Neuroscience, Anatomy Building, University of Cambridge, Cambridge, United Kingdom; 2 Department of Ophthalmology, Addenbrookes Hospital, Cambridge University Hospitals NHS Trust, Cambridge, United Kingdom; 3 Vision and Eye Research Unit (VERU), Anglia Ruskin University, Cambridge, United Kingdom; LAAS-CNRS, FRANCE

## Abstract

The purpose of this study was to evaluate the effects of vital dyes on human Descemet's membranes (DMs) and endothelia. DMs of 25 human cadaveric corneas with research consent were treated with dyes routinely used in Descemet membrane endothelial keratoplasty (DMEK), 0.05% Trypan blue (TB) or a combination of 0.15% Trypan blue, 0.025% Brilliant blue and 4% Polyethylene glycol (commercial name Membrane Blue Dual; MB). The effects of these two dyes on (i) endothelial cell viability, (ii) DM mechanical properties as assessed by atomic force microscopy, and iii) qualitative DM dye retention were tested for two varying exposure times (one or four minutes). No significant differences in cell toxicity were observed between treatments with TB and MB at the two different exposure times (P = 0.21). Further, both dyes led to a significant increase in DM stiffness: exposure to TB and MB for one minute increased the apparent elastic modulus of the DM by 11.2% (P = 8*10^−3^) and 17.7%, respectively (P = 4*10^−6^). A four-minute exposure led to an increase of 8.6% for TB (P = 0.004) and 13.6% for MB (P = 0.03). Finally, at 25 minutes, the dye retention of the DM was considerably better for MB compared to TB. Taken together, a one-minute exposure to MB was found to improve DM visibility compared to TB, with a significant increase in DM stiffness and without detrimental effects on endothelial cell viability. The use of MB could therefore improve (i) visibility of the DM scroll, and (ii) intraoperative unfolding, enhancing the probability of successful DMEK surgery.

## Introduction

**Fig 1:**
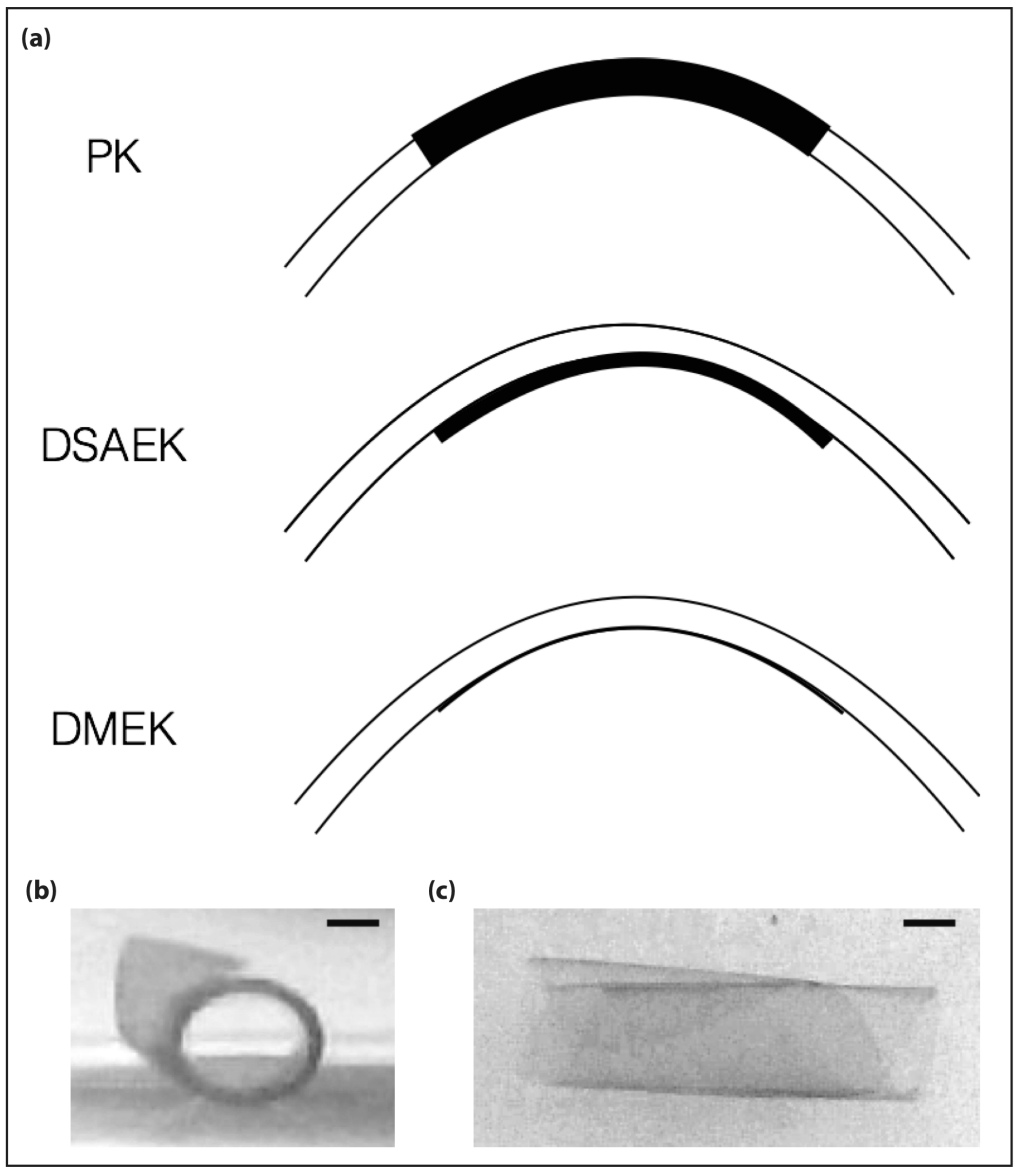
Keratoplasty. (a) Schematic drawings of different approaches to the transplantation of corneal tissue. PK = Penetrating keratoplasty, DSAEK = Descemet stripping automated endothelial keratoplasty, DMEK = Descemet membrane endothelial keratoplasty.). (b) Side view and (c) top view of human DM in buffer solution. DM tissue is transparent and tends to coil up, posing intraoperative challenges to unfolding during DMEK surgery. Representative grey-scale images of coiled up 8 mm DM scroll. Scale bar = 1 mm.

Corneal transplantation also referred to as ‘keratoplasty’, is the mainstay of treatment for corneal endothelial disorders which lead to visual compromise [1]. Originally, full-thickness corneal tissue (‘full thickness penetrating keratoplasty’, PK) was transplanted. The introduction of posterior lamellar keratoplasty—i.e. the selective transplantation of specific lamellar corneal tissue layers—by Melles et al. in 1998 [2–5] (Fig 1A) revolutionised the field and significantly improved postoperative visual acuity results. Since then, this technique has undergone further refinements, culminating in the two currently most commonly performed procedures (a) Descemet stripping automated endothelial keratoplasty (DSAEK), and (b) Descemet membrane endothelial keratoplasty (DMEK) [6–9].

DMEK refers to the replacement of the complex of Descemet’s membrane (DM) and the underlying endothelium. It leads to faster and better visual recovery for patients with up to 77% of eyes achieving a best-corrected visual acuity (BCVA) > 20/25 at six months, and with most patients attaining this already by the end of three months [10]. Furthermore, DMEK has the advantage of reduced allograft rejection rates (<1%) compared to PK (5–15%) and DSAEK (10%) [11–13].

However, donor tissue preparation, visualisation, and intraoperative handling pose significant challenges to surgeons; DMs are transparent and tend to coil up [14] (Fig 1B and 1C). Hence, vital dyes are used to stain and visualise the DM and endothelial cells [15] and play an essential role during the surgical process, helping to achieve a congruous anatomical attachment of the transplant to the host posterior corneal stroma. 0.05% RS Trypan Blue (TB; ALCHIMIA S.r.l, Austria) has been used in DMEK with reasonable success. However, in a subset of cases repeated application of the dye is required to maintain tissue staining and DM visibility during surgery. In current surgical practice, a dye that stains the transplant for the whole duration of the operation, while keeping damage to the tissue to a minimum, and ideally temporarily stiffening the membrane to minimise its inherent spiralling properties, is needed.

Recently, a vital dye termed Membrane Blue Dual (MB), composed of 0.025% Brilliant Blue (BB), 0.15% TB, and 4% polyethylene glycol (PEG) (D.O.R.C. Dutch Ophthalmic Research Centre International B.V) has been introduced for the use in vitreoretinal surgery [16]. However, the effect of the increased dye concentration and the addition of BB and PEG in MB on the human corneal endothelium, and its performance compared to the current standard dye TB are currently poorly understood.

**Fig 2:**
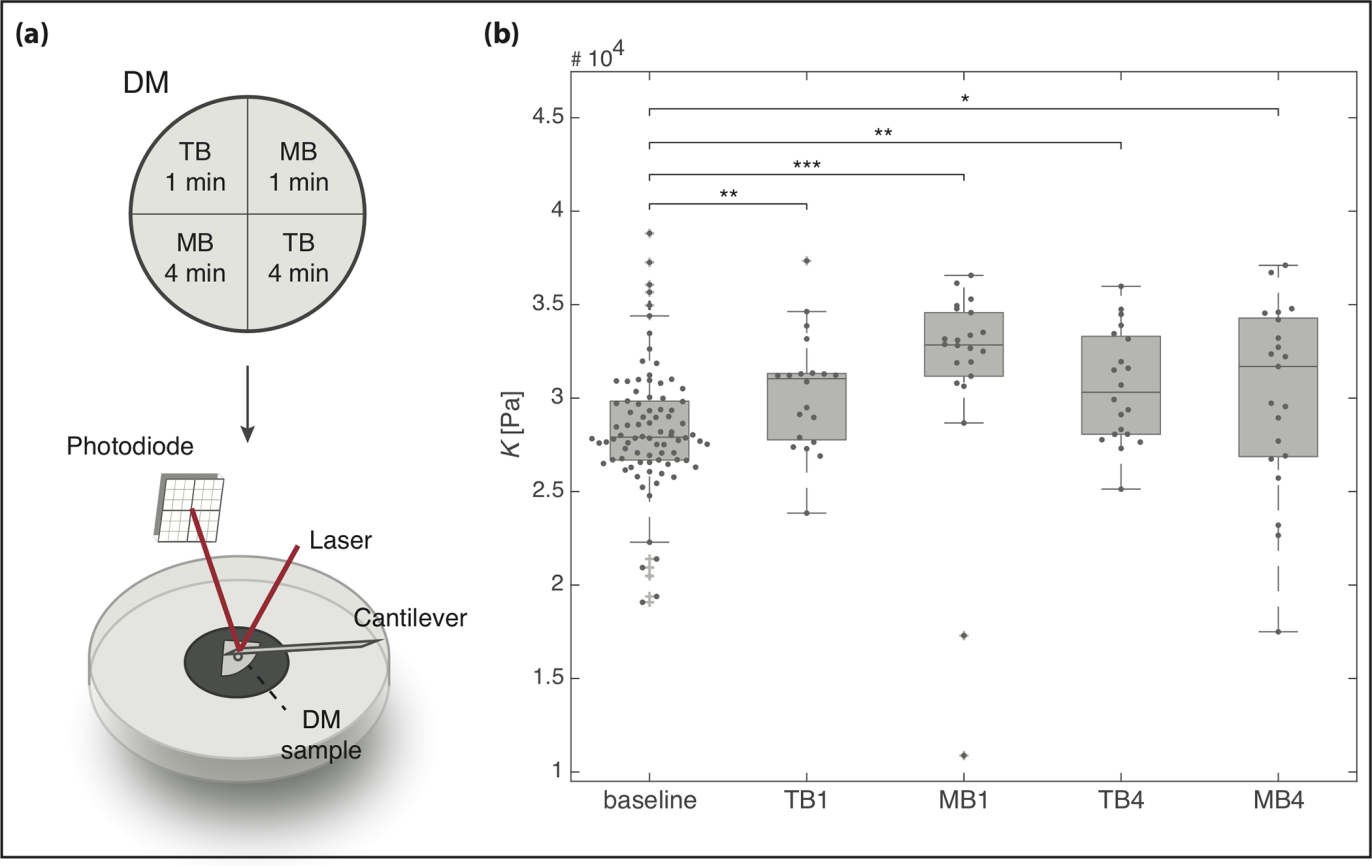
Assessment of changes in DM elastic moduli using AFM. (a) Schematic drawing of the experimental setup. Each cornea was cut into four pieces. AFM measurements were performed before exposure to a vital dye (providing a baseline *K* value) and one or four minutes after exposure to TB (TB1, TB4) or MB (MB1, MB4). (b) Boxplots showing apparent elastic moduli *K* of the four different groups. All treatments led to a significant increase in corneal stiffness (n = 3, P < 0.001, Kruskal-Wallis Anova). Treatment with MB led to a slightly larger but non-significant increase in tissue stiffness compared to TB. Dark lines indicate median values, grey boxes the Q1-Q3 percentiles. (* (P < 0.05); ** (P < 0.01); *** (P< 0.001), Wilcoxon rank sum test). DM = Descemet’s Membrane, TB = RS Trypan Blue, MB = Membrane Blue Dual.

To address this gap, we compared the effects of TB and MB on human DM tissue, evaluating (i) endothelial toxicity and (ii) biophysical properties, i.e. tissue elasticity, for two different exposure times (one and four minutes), and (iii) staining retention measured after 60 second exposure to either of the two dyes. To quantify tissue elasticity, we exploited atomic force microscopy (AFM): a soft leaf spring (‘cantilever’) is used to push on the tissue with a controlled force, and deformation is measured (Fig 2A). The relation between applied force and resulting indentation can be used to extract an apparent elastic modulus (K). AFM has been previously used to assess mechanical properties of biologic materials [17–19] and the individual layers of the human cornea [20–21].

## Methods

### Human corneal tissue

A total of 25 human cadaveric donor corneas from 24 deceased donors were included in this study. Human cadaveric corneas were procured from the national eye banks UK with consent for research and material transfer agreement reviewed and project approved by Bristol Eye Bank, University of Bristol, U.K. The experiments were undertaken in HTA approved research facility (12315). The study adhered to the principles in the Declaration of Helsinki. None of the tissue donors were from a vulnerable population and all donors or next of kin provided written informed consent for use of corneas for research purposes. The donor corneas had an age range of 35 to 75 years (mean = 65y +/- 16y) and consisted of 13 males and 11 females. The corneas were held in organ culture storage for an average 30 +/- 7 days prior to experiments and had a mean endothelial density of 2102 +/- 160 cells /mm^2^.

### Assessment of corneal endothelial cell viability

**Fig 3:**
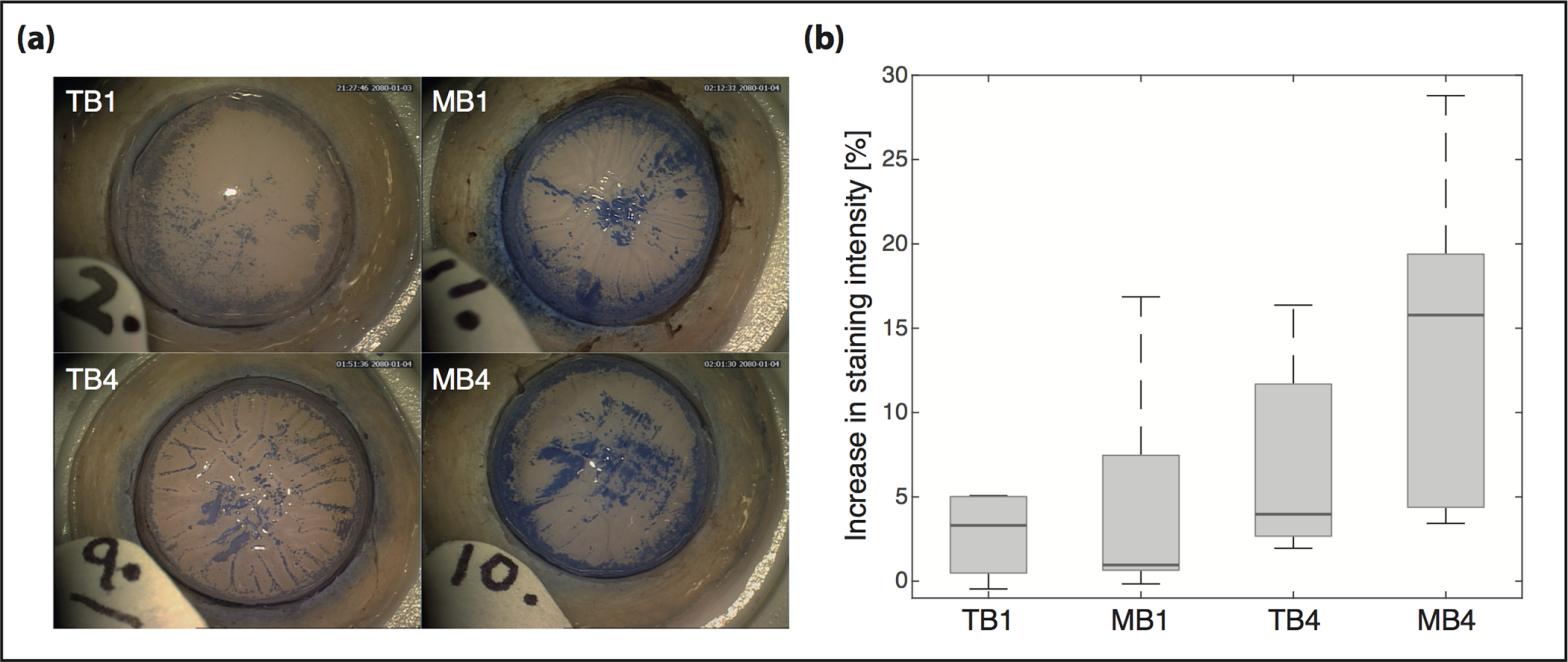
Assessment of TB and MB toxicity on human corneal endothelium in human corneas. (a) Images of corneal tissue after one and four minutes of exposure to the two dyes (RS- Blue,TB and Membrane blue Dual MB). (b) Boxplot showing the relative increase in staining intensity after exposure to TB and MB for one and four minutes (n = 5; P = 0.2115, Kruskal-Wallis ANOVA). Dark lines indicate median values, grey boxes the Q1-Q3 percentiles.

20 cadaveric human donor corneas were randomised into four groups (n = 5). There was no significant difference in age of donors between the groups. To stain the corneal endothelium, each cornea was removed from Eagle’s medium (Sigma, UK) and placed in a balanced salt solution (BSS, Alcon, UK) for 30 seconds. Thereafter, the tissue was mounted onto a holder (Coronet, Network Marketing Ltd, UK), endothelium facing up, and 0.5 ml of TB was applied for one minute. The dye was subsequently removed using a weckcell sponge, and the tissue was rinsed with balanced salt solution (BSS). Using a Zeiss Lumera 300 surgical microscope (Carl Zeiss Meditec AG), a baseline image of the endothelial cellular staining was recorded at a fixed luminance and magnification. Subsequently, the corneas were exposed to one of the respective vital dyes (TB and MB) for either one (TB1, MB1) or four minutes (TB4, MB4). The corneas were then rinsed with BSS and a second image was recorded (Fig 3A)

Image processing was undertaken using MATLAB 2014a with Image Processing Toolbox (MathWorks, Natick, USA). The resolution of the captured images was 768 x 576 pixels. The magnification was set such that the full diameter of the cornea occupied approximately the central 500 pixels, and a measure was included in several images to ensure no change in magnification had occurred. Lighting levels were kept constant throughout all data collection. In each image, the location of the corneal limbus was determined at 12, 3, 6 and 9 o’clock. These points were used to ascertain the centre of the corneal endothelium by mathematical averaging. A disc equivalent to 8 mm of the central corneal endothelium was cut out from each raw image in MATLAB. The discs were converted from colour to black-and-white images [22] using a threshold procedure. The threshold was set such that stained areas of endothelium were represented as black and unstained areas were represented as white. The amount of staining in each image was determined by expressing the number of stained pixels as a percentage of the total number of pixels in each disc. The increase in staining caused by each intervention could then be determined for each cornea and quantified as a metric for endothelial damage induced by the intervention.

### Preparation of Descemet’s membrane for atomic force microscopy and dye retention

Human cadaveric corneas were placed endothelial side up on corneal holders with 0.5 ml BSS. The peripheral DM and trabecular meshwork margin was identified under a surgical microscope (Zeiss Lumera 300) and scored 360 degrees circumferentially using a Sinsky hook. Subsequently, the edge of the DM was lifted off the posterior corneal stroma and manually peeled off centripetally with non-toothed surgical tissue holding forceps, leaving only a central attachment. Following this, an 8 mm corneal trephine (Coronet, Network medicals, UK) was centred on the endothelium and under gentle application of pressure the central circular DM measuring 8 mm in diameter was peeled off. The DM scroll was stored in BSS for subsequent experimentation.

### Atomic force microscopy (AFM)

Three Descemet membranes (central 8 mm) from three different human donor corneas (Mean age 60 yrs) were cut into four pieces, as shown in Fig 2A, resulting in 12 samples. The individual DM samples were subsequently mounted with the DM facing up (and thus the endothelial layer facing away from the cantilever) on BD Cell-Tak-coated (Cell and Tissue Adhesive; BD Biosciences) cell culture dishes (TPP) and covered with BSS. For each sample, five to nine force-distance curves were taken at a maximum force of 100 nN (approach speed = 10 μm/s) using a JPK Cellhesion 200 AFM (JPK Instruments AG, Berlin, Germany) set up on an inverted optical microscope (Axio Observer.A1, Carl Zeiss Ltd., Cambridge, UK) as baseline values. Tipless silicon cantilevers (Sicon-TL, Nano World, Neuchatel, Switzerland; spring constant ~0.2–0.3 N/m) were custom-modified by attaching polystyrene beads (d = 37 μm; micro-particles GmbH, Berlin, Germany) as probes. To avoid inter-corneal variability the four samples from each cornea were subsequently stained with one of the two dyes for either one (TB1, MB1) or four minutes (TB4, MB4), rinsed with PBS and again probed by AFM. To assess the change in elastic modulus after incubation, the data was subsequently fit to the Hertz model [23] using a custom-written algorithm [24]:

$$F=\frac{4}{3}\;\frac{E}{1-v^{2}}r^{1/2}\delta^{3/2}=\frac{4}{3}Kr^{1/2}\;\delta^{3/2}$$

Where ***F*** is the applied force, ***E*** the Young’s modulus, *v* the Poisson’s ratio, ***r*** the radius of the probe, ***δ*** the indentation depth, and $K=\frac{E}{1-v^{2}}$ the apparent elastic modulus. All curves were analyzed at an indentation depth of 1 μm.

As Trypan blue is a photosensitizer, which might lead to slight changes in tissue stiffness after light exposure, we kept the light intensity constant throughout all experiments and exposed all samples to light for the same amount of time.

### Comparison of staining intensities

Two 8 mm DM samples from a 66 yrs old male donor were exposed to 0.5 ml of either TB or MB for 60 seconds. The two DM scrolls were subsequently washed in a petri dish containing 20 ml of BSS. Serial photographs of the DM scrolls were recorded over a period of 25 minutes. For qualitative comparison of staining intensities, images were converted to grey scale and a Fourier Filter was applied to correct for irregular illumination.

## Data analysis

Statistical analysis was done using MATLAB software. The increase in corneal endothelial staining (non-viable cells) upon exposure to vital dyes at different times was calculated as % increase in staining. Baseline data from AFM measurements were pooled across the four different groups. The significance of data was tested using the Wilcoxon rank sum test and the Kruskal-Wallis ANOVA test. Significance levels are *P < 0.05; **P < 0.01; ***P < 0.001.

## Results

### Endothelial cell toxicity upon dye exposure

In order to determine potential differences in cell toxicity between the two dyes, we analysed the change in staining pattern of the endothelial cells from baseline of DMs treated with either dye. No significant difference between the four groups (TB1, TB4, MB1, MB4; P = 0.2115, Kruskal-Wallis, ANOVA) was observed after staining the corneal endothelium with either of the two dyes. After exposure to TB, the median percentage increase in endothelial cell staining was found to be 3.3% after one, and 4.0% after four minutes. Exposure to MB led to a median increase in staining intensity of 1.0% after one, and 15.8% after four minutes (Fig 3B).

### Change in elastic modulus of Descemets membrane upon vital dye exposure

We quantified the elastic stiffness of corneal tissue before and after treatment with different dyes using AFM. Exposure to both vital dyes at one and four minutes led to a significant increase in DM elastic modulus compared to baseline values. The median baseline elastic modulus values extracted from stiffness measurements of the 12 individual DM pieces (TB1, MB1, TB4, MB4; three samples per condition) measured before dye exposure was 27908.5 Pa SD = 3439.7 Pa. Following TB exposure for one and four minutes, we observed a median increase in elastic modulus of 11.2% and 8.6%, respectively, compared to baseline (P = 0.0082 and P = 0.0041, respectively). Similarly, MB exposure for one and four minutes led an increase in DM stiffness of 17.7% and 13.6%, respectively (P = 0.000004 and P = 0.03, respectively) (Fig 2B).

### Assessment of dye retention

**Fig 4:**
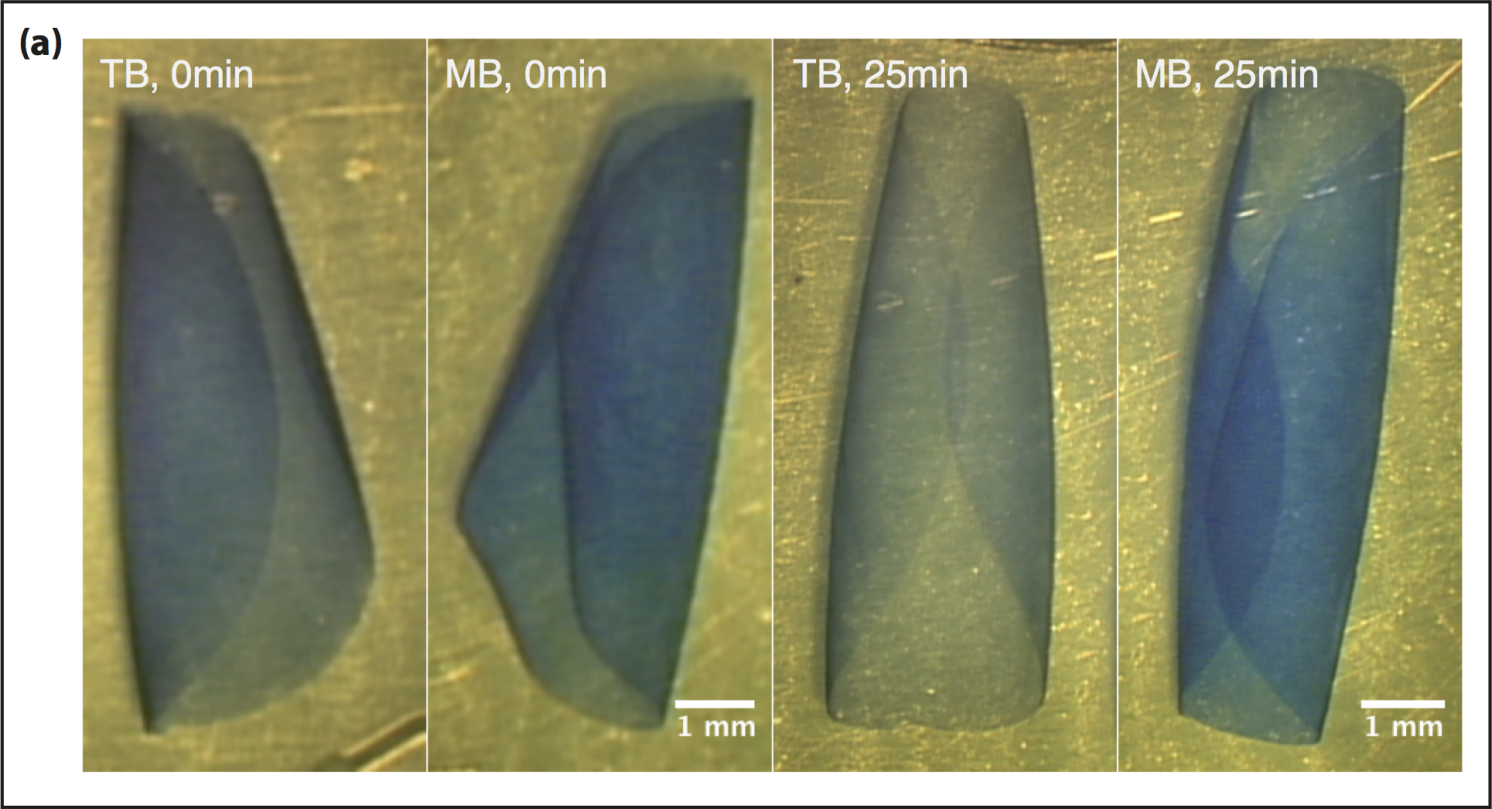
Retention of dye after 25 min treatment. (a) Image of Descemet membranes stained with Trypan Blue (TB, left) and Membrane Blue (MB, right). Dye retention was assessed 25 min after staining. (Scale bar = 1 mm).

Finally, we tested the dye retention times of TB and MB after exposure to 0.5 ml TB or MB. While TB staining waned gradually, MB-treated DMs retained the dye effectively at 25 minutes. (Fig 4)

## Discussion

There is a constant drive to refine the surgical technique DMEK to minimise intraoperative complications and improve transplant attachment to the host posterior corneal stroma [1–2]. Adequate visibility of DM scrolls during surgery is a key factor to achieve unfolding and accurate placement of the transplant [25–26]. For this purpose, vital dyes, in particular, Trypan Blue (TB) and to a lesser extent Brilliant Blue (BB)[25], have been used. However, the time- and concentration-dependent effects of such dyes on corneal endothelial toxicity have not been fully evaluated. To promote the use of vital dyes in DMEK, this information would be essential. In a recent study, Majmudar et al. showed that staining of human DM tissue with TB for up to 3 min led to adequate staining for prolonged periods of up to 130 min, without any effect on endothelial cellular viability [27].

Assessing the performance of the two vital dyes TB and MB, we observed moderate levels of endothelial cell death following exposure to either dye, both at one and four minutes. However, between the two dyes, no significant difference in endothelial cell viability was observed. The general increase in endothelial cell death upon vital dye exposure [27] needs to be borne in mind during DMEK surgery, and prolonged or repeated exposure to the dyes should be avoided.

MB, which is a combination of BB, Polyethylene glycol (PEG) and TB, showed improved DM staining properties; a single exposure to MB for one minute was sufficient to stain DMs over a period of 25 minutes, a time window relevant for DMEK surgery. The superior tissue staining properties of MB could circumvent the need for repeated dye exposure that is required in certain DMEK cases when using TB, therefore avoiding unnecessary cell loss and provide better visibility of the DM scroll.

When we compared the effect of exposure to either TB or MB on the elasticity of the 10–20 μm thick human DM, we observed a significant stiffening of up to 17.7% (median elasticity of MB1 compared to baseline). The above results are in line with recent reports, in which DMs were exposed to TB and assessed by AFM; a 60-second exposure to 0.06% Trypan blue led to increased rigidity of DMs [26–28]. In their study, the authors had highlighted the need to assess the time-dependent effects of vital dyes on human DMs, which we now present. Our study is the first to compare the time-dependent effects of vital dye exposure (one minute vs. four minutes) of not only 0.05% Trypan blue, but also of a combination of 0.15% TB, 0.025% BB and 4% Polyethylene glycol (PEG) on human DM stiffness. We argue that the stiffness modifying effect of vital dyes on DM could have a clinical benefit, as this might facilitate the unfolding of the tight DM scrolls, particularly common for young corneal donors (age < 55 years), as these tend to form tight rolls [29]. The number of samples included in the study was limited, as availability of human corneal tissue for research is finite. Randomization and diligent use of the samples allowed us to minimise the donor age-dependent bias and inter-observer variations.

**Fig 5:**
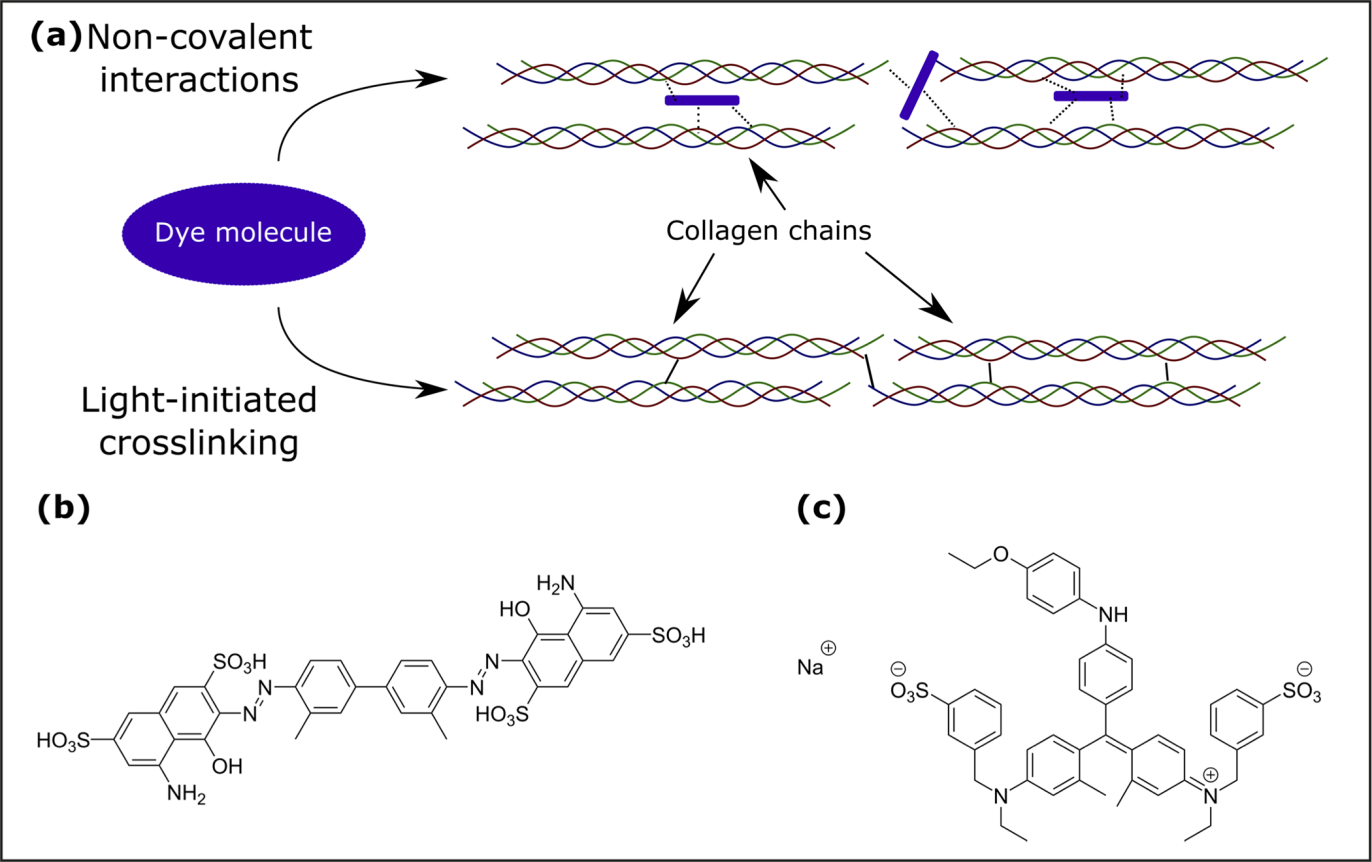
Possible mechanisms of corneal stiffening by TB and MB. (a) The two possible modes of crosslinking of the ECM. Dye intercalates the protein structure of the ECM in the non-covalent mode, increasing the number of attractive supra-molecular interactions (dashed lines). Photochemical excitation of the dye, on the other hand, could lead to the generation of covalent crosslinks (solid lines) between nearby amino acid residues on the proteins. (b) Trypan Blue C) Coomassie Brilliant Blue G 250. Both dyes contain a variety of polar and non-polar functional groups, allowing for multiple kinds of non-covalent interactions.

The mechanisms involved in increasing DM tissue stiffness after dye exposures are presently unknown. The human DM is rich in collagen (specifically in collagen types IV and VIII) [30–32] and the increase in DM stiffness could result from physical and/or chemical crosslinking of the ECM. Therefore, we speculate that physical crosslinking could occur through supra-molecular interactions between the dye molecules and the surrounding proteins (Fig 5A). Both, TB and MB, have multiple functional groups that make them suitable for such interactions (Fig 5B and 5C). Chemical crosslinking of the collagen ECM occurs through the photochemical generation of radical species in the solution by photosensitization with the dye. Excitation of the dye molecules with light causes them to form radical intermediates on the accessible surfaces of the protein fibres. Those intermediates can then potentially react with each other in an interfibrillar fashion (Fig 5A). In agreement with this hypothesis, Wollensak et al. observed an increase in crosslinking of the collagen matrix of the anterior lens capsule under TB treatment when exposed to light. This was accompanied by an increase in lens stiffness [33]. No such increase in stiffness was found in the absence of light, suggesting a dominant role of photochemical mechanisms. Haritoglou et al. screened the mechanical effect of multiple dyes on lens capsule stiffness, including TB and Coomassie BB (which is found in MB but not in TB) [34]. Both molecules led to an increase in lens stiffness already in the absence of light, with a further increase in stiffness upon irradiation. Coomassie BB resulted in a significantly larger increase in stiffness than TB [34]. Its strong affinity for binding to some proteins [35], including collagen IV [36], could point to the origin of the slightly larger stiffening of MB-treated corneas compared to TB treated ones. Furthermore, MB contains more TB (0.15%) than most commercially available TB dyes (0.05 to 0.06%). The higher concentration of trypan blue in MB could also have contributed to (i) the enhanced staining (Fig 3) and (ii) the tissue stiffening (Fig 2) observed in our study. The vital dye induced tissue staining of DM is recognised to be a temporary effect with complete recovery of DM transparency within a few days but whether the same applies to the change in elastic modulus of the membrane requires future assessment.

In Summary, our data demonstrates that the combination of Trypan blue 0.15% with BB and PEG (MB) that could have a significant clinical benefit in DMEK. MB treatment prior to DMEK (i) improves the visibility of the DM during surgery, and (ii) increases its stiffness, which we believe to be beneficial for the quick unrolling of the tissue during surgery, ultimately enhancing anatomical and functional success rates of DM transplantation.
